# VIRTUAL REALITY IN RESPITE: USING IMPLEMENTATION SCIENCE TO ASSESS ADOPTION OF VR WITH PLWD

**DOI:** 10.1093/geroni/igae098.2572

**Published:** 2024-12-31

**Authors:** Mary Milnamow, Lillian Agyemang, Louanne Bakk, Jason Dauenhauer

**Affiliations:** University at Buffalo, Buffalo, New York, United States; University at Buffalo, SUNY, Buffalo, New York, United States; University at Buffalo, SUNY, Buffalo, New York, United States; State University of New York at Brockport, Brockport, New York, United States

## Abstract

In the U.S., 6.7 million older adults are living with dementia (PLwD); this number is estimated to double by 2040. Community-based volunteer respite programs are vital for PLwD to receive social connections and engagement. Virtual Reality (VR) programming for PLwD is typically used in nursing homes, residential care, and rehabilitation settings. Few studies have examined using VR with PLwD and caregiver dyads receiving community- and home-based services. This study applied Implementation Science to identify facilitators and barriers in adopting a VR intervention within a respite program and to develop evidence-based strategies to guide successful implementation. The RE-AIM framework evaluated changes in VR skills, confidence, comfort, and buy-in from respite staff and volunteers. Electronic surveys were completed anonymously before VR training and after completing one VR session. The IAM and FIM were also used to assess the intervention’s acceptability, appropriateness, and feasibility. After training and practicing VR, a paired samples t-test revealed that participants significantly improved across all areas, with the most significant improvement in VR skills from pre to post (16.36 (1.31) vs. (28.00 (1.26), t= -4.44, p=.0001, respectively). The strongest facilitator was the practical and financial support provided by leadership. A persistent challenge was the cost of VR technology. Implementation strategies contributing to successful VR programming included identifying and training champions and developing a trauma-informed quick guide. Findings demonstrate effective utilization of Implementation Science to address the strengths and weaknesses of incorporating a novel intervention that prepares staff, volunteers, and families to experience VR adventures.

